# Environmental Noise Classification Using Convolutional Neural Networks with Input Transform for Hearing Aids

**DOI:** 10.3390/ijerph17072270

**Published:** 2020-03-27

**Authors:** Gyuseok Park, Sangmin Lee

**Affiliations:** Department of Electronic Engineering, Inha University, Incheon 22212, Korea; gyuseok.park@gmail.com

**Keywords:** hearing loss, hearing aids, environmental noise, deep learning, convolutional neural networks

## Abstract

Hearing aids are essential for people with hearing loss, and noise estimation and classification are some of the most important technologies used in devices. This paper presents an environmental noise classification algorithm for hearing aids that uses convolutional neural networks (CNNs) and image signals transformed from sound signals. The algorithm was developed using the data of ten types of noise acquired from living environments where such noises occur. Spectrogram images transformed from sound data are used as the input of the CNNs after processing of the images by a sharpening mask and median filter. The classification results of the proposed algorithm were compared with those of other noise classification methods. A maximum correct classification accuracy of 99.25% was achieved by the proposed algorithm for a spectrogram time length of 1 s, with the correct classification accuracy decreasing with increasing spectrogram time length up to 8 s. For a spectrogram time length of 8 s and using the sharpening mask and median filter, the classification accuracy was 98.73%, which is comparable with the 98.79% achieved by the conventional method for a time length of 1 s. The proposed hearing aid noise classification algorithm thus offers less computational complexity without compromising on performance.

## 1. Introduction

Hearing difficulty is a symptom of hearing loss caused by anomalies in the human sound signal transmission process. The difficulty in hearing a particular sound is due to an increase in the corresponding hearing threshold and narrowing of the dynamic range [1,2]. The use of a hearing aid is one of the methods for solving hearing difficulty and compensating for hearing loss [3]. Hearing aids use various technologies such as noise reduction, sound compensation, directional microphones, and feedback cancelation, and are tuned to the hearing characteristics of the users and the environments of use [4].

Daily life is full of noises, and hearing aid technologies are continuously being developed to reduce these noises, such as those in restaurants, car horns, buzzing from electrical equipment, and random voices in the surroundings. However, because the operating environment of a hearing aid varies with time, place, and other factors, 100% performance satisfaction is not achieved [5,6]. One of the biggest complaints of hearing aid users is the inability to completely reduce ambient noise, and the tendency of the noise to be amplified with the human voice [7]. Speech intelligibility is affected when surrounding noise is incorrectly interpreted as human voice, or if the voice is misinterpreted and removed with noise. This is due to performance problems and inaccurate sound classification of the noise reduction algorithm [8].

The traditional noise classification algorithm in hearing aids proceeds with the extraction of characteristic features from the data, finding the class with the highest probability based on those features, and classifying them based on the identified class [9]. The noise classification algorithm mainly focuses in the performance of the hearing aid, which has to operate with a low computational complexity and low power [10].

However, with the recent development of hearing aid chips with early smart phone-level CPU performance, such as Ezioro 71XX, the use of environmental noise classification algorithms that use deep learning is now feasible. It is generally not easy to extract sound signal characteristics that can be used as input data for deep learning, compared with image signals. This is because the time-domain data of sounds are difficult to know with respect to their signal information or their characteristics in the frequency domain. Therefore, various feature extraction algorithms are used to switch the data into the frequency domain and to distinguish the frequency characteristics of the different sounds. Nevertheless, real sound signals are a mixture of different sounds, and it remains difficult to distinguish between the characteristics of the contained noises and voices.

In this study, a noise signal spectrogram was used to transform sound signals in the time frequency-domain into image signals for hearing aid noise classification, as an alternative to the use of extracted frequency-domain features. The long noise estimation period was employed, and deep learning was used to improve the low classification accuracy. The image data transformed from the sound signals were particularly used in the present study for the classification of environmental noises with the aid of convolutional neural networks (CNNs), which are some of the best methods for image classification.

## 2. Previous Research

### 2.1. Conventional Noise Classification Algorithms

One of the most basic classification algorithms in use is the Bayesian classifier [11]. It classifies with the help of histograms of the class-specific probabilities. The K-nearest neighbors classification algorithm is a simple process that determines the class of a new input [12]. It is suitable for simple classification problems with relatively few training features, because, as the number of training feature increases, both the computational complexity and time increase. Support Vector Machine [13,14] and Neural Networks [15] are discriminative classification algorithm. These algorithms can be effective when there is enough sufficiently varied data to train the classifier, and can work even in those situations where the underlying probability distributions for the features are unknown. Hidden Markov models [10,16,17] are a widely used statistical method for speech recognition. One major advantage of HMMs over the previously described classifiers is that they account for the temporal statistics of the occurrence of different states in the features. Clustering refers to a group of unsupervised processes that group features based on their measured similarity. Clustering is related to classification in that both divide unknown inputs into classes. 

### 2.2. Convolutional Neural Networks

**Figure 1:**
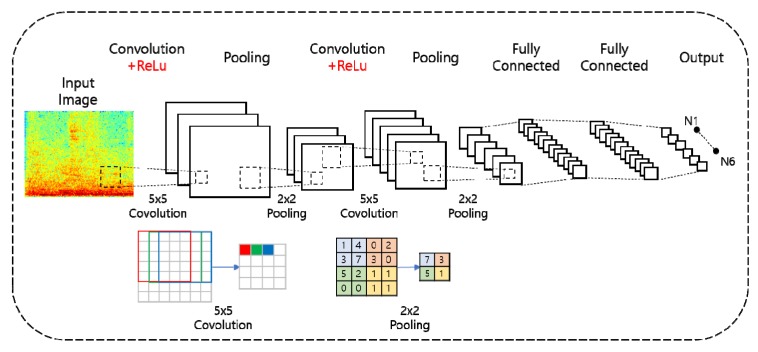
The structure of Convolution Neural Networks.

A CNN is a deep learning technology based on supervised learning, and is widely used for image processing while maintaining the spatial information of the image [18,19]. As shown in Figure 1, convolutional and pooling layers were added between the input and output layers of the present CNN, for excellent performance in processing data composed of multi-dimensional arrays such as color images. The convolution work is for extracting the high-level features such as edges, from input data. Similar to the convolutional layer, the pooling work is responsible for reducing the spatial size of the convolved feature [20]. This is to decrease the computational power required to process the data through dimensionality reduction.

The feature map of the input data is produced by moving a convolution filter in the convolutional layer, and the values obtained from the final feature maps are then extracted to reduce the computational complexity and improve the accuracy of the pooling layer [21].

In this paper, the CNN has two hidden layers, namely the 5 × 5 convolution layer and max pooling layer, which uses a 2 × 2 window. The activation function is a ReLu function, which is the most commonly used function, and the loss function is a cross-entropy function. The overall data was divided into training and test sets at a ratio of 75:25, respectively, the batch sizes of the training was set to 16. The number of epochs was 12 and learning rate was set 0.001.

## 3. The Proposed Algorithm

In this paper, the spectrogram images of noise signals were used as input data for the CNNs, without feature extraction or conversion to the frequency domain. A spectrogram is a visual representation of the frequency spectrum of the signals with respect to time. The amplitude of the sound frequency was indicated by color in the spectrogram. Because the spectrogram consisted of different image colors, it had the advantage of enabling verification of the time and energy information in the frequency domain over a certain period. Therefore, the characteristics of the spectrogram images varied with the amplitude of the frequency and the time information.

**Figure 2:**
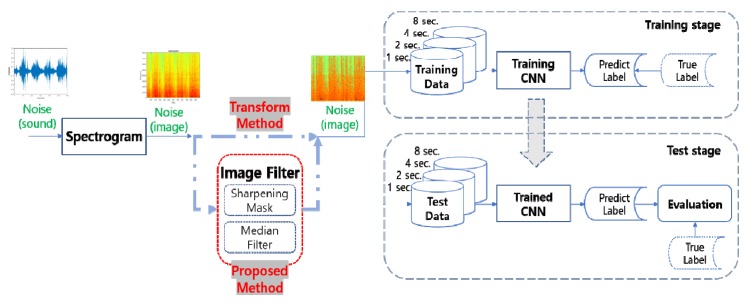
The process of the noise classification with convolutional neural networks by the proposed algorithm.

Figure 2 shows a flow chart of the proposed environment noise classification algorithm. Generally, the input sound signal data were transformed into spectrogram images represented as RGB colors for noise classification using the CNNs. Two types of filters were combined and used to distinguish the noise characteristics, because the spectral image of the sound signals contained irregular amplitude changes over time unlike normal image signals. Each filter was introduced and applied to compare the results of the proposed algorithm in the process.

The first image filter uses a sharpening mask (method #1: spectrogram + Sharpening Mask), which enables enhancement of the boundaries of the noise characteristics [22]. The filter clearly identifies the boundaries of the colors, so that the area of the high-energy noise signals can be more clearly displayed. 

The second image filter uses a median filter (method #2: spectrogram + Median Filter) [23]. In a conventional noise signal spectrogram, there are irregular low-energy pixels between the noise feature pixels that appear red. The use of the median filter compensates for these low-energy pixels when the data is used as input for the CNNs. Sets of input data with four different time lengths (Sets A, B, C, and D) were fed to the CNNs, and the corresponding noise classification accuracies were compared.

## 4. Materials and Methods

The conditions of noise data and signal processing information will first be discussed in Section 4.1; in Section 4.2, the specific noise classification experiment and input data transformation process will be described. Overall, the determination of the input data and detailed pre-processing of image data in CNNs are described for the noise classification algorithm.

### 4.1. Recording Environmental Noises

Ten kinds of noise were recorded from real environments in which hearing aids are used: white noise (white, N0), café noise around Inha University, Korea (café, N1), interior noise in a moving car (car_interior, N2), single fan noise in a laboratory (fan, N3), laundry noise in a laundry room (laundry, N4), noise in the library of Inha University (library, N5), normal noise in a university laboratory (office, N6), various noises in a restaurant (restaurant, N7), noise in subway car (subway, N8), and traffic noise around an intersection (traffic, N9). 

Each noise was recorded three times at different times on different weekdays, and noise data for each noise type was generated for 30 min. To be closely related to the hearing aid’s environment, recording places were selected such as Starbucks, the biggest restaurant in the Inha Student Union building, etc. The noises were recorded on an iPhone 6S at 44.1 kHz, which is the highest possible sampling frequency, and the artificial noise generated at the beginning and end of recording was not included. The noise data was subsequently down-sampled to 16 kHz, which is the proper frequency for signal processing for hearing aids.

### 4.2. Experiment Data

The Matlab R2019b program developed by MathWorks was used to divide the recorded noise data into certain time intervals. The noise signals consisted of 16,000 samples per second, divided into four sets with time lengths of 1.0, 2.0, 4.0, and 8.0 s, respectively. Each frame was overlapped by 25% on either side to achieve a continuous noise signal and prevent to loss some data [24]. The spectrogram images obtained from the sound signals consisted of 23,960 images with a time length of 1 s (Set A), 11,960 of length 2 s (Set B), 6,000 of length 4 s (Set C), and 3,000 of length 8 s (Set D) in each of the 10 noises.

**Figure 3:**
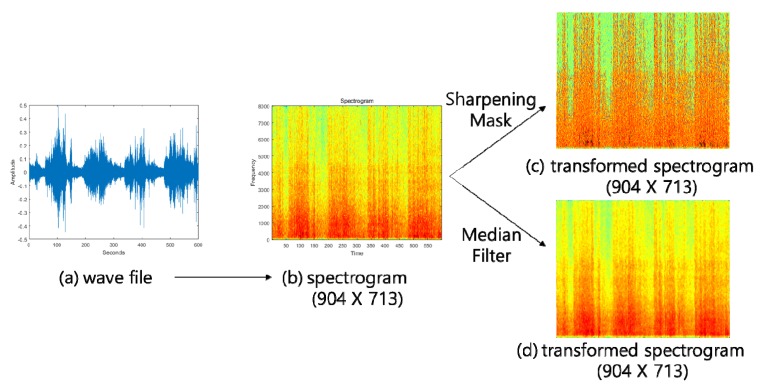
The transformed spectrogram image (**b**) from the noise signal (**a**), and when applying the sharpening mask (**c**) and the median filter (**d**).

The conversion functions in the signal processing tool in the Matlab Toolbox was used to transform the noise signals into a spectrogram. The spectrogram image had a resolution of 904 × 713 pixels and was used as the input of the CNNs. To increase the classification accuracy of the spectrogram images of the noise signals, a 3 × 3 sharpening mask was used to enhance the boundaries of the colors, while a 5 × 5 median filter was used to clearly represent the pattern of the colors and make a color smoothing for random noise pixels. Figure 3 shows the results of a transformation of sound signals into a spectrogram image and the application of the sharpening mask and median filter. The spectrogram images were also subsequently used as input data.

Using Set A as an example, there were four types (spectrogram image, spectrogram image + Sharpening Mask, spectrogram image + Median Filter and spectrogram image + Sharpening Mask + Median Filter) of input data for each of the 10 considered environments, from which 23,960 spectrogram images were obtained. The same number of images were obtained after the application of the sharpening mask and median filter, respectively.

## 5. Experimental Results

In this section, results of the classification for hearing aids are introduced with various conditions, showing the detailed performance as proposed algorithms. Results are presented as a Confusion Matrix and a Receiver Operating Characteristic (ROC) curve. A Confusion Matrix is a table that is often used to describe the performance of the classification on a set of test data [25]. A ROC curve is a graph showing the performance of a classification model at all classification thresholds [26].

### 5.1. Performance Evaluations

This section presents experimental results of the proposed environmental noise classification for hearing aids using a CNNs. The classification produced varying results because the noise signals were randomly divided into training (0.75) and test (0.25) sets, and the spectrogram images corresponded to different times. The total number of input data was 5990 when the length of time was 1 s, and the number of test data in each noise was 599. Because the number of input data was dependent on length of time, the number of test data is 2990 in 2 s, 1500 in 4 s, and 750 in 8 s. 

In order to show significant classification results, every experiment of training set and test set were randomly divided at a constant rate. As indicated in Table 1, the values in the tables are the classification accuracy (%), which is the ratio of number of correct predictions to the total number of input data, and the noise classification was performed 10 times. Bold numbers in the bottom two rows of each table are an average and a standard deviation of classification accuracies for comparing with other conditions. The conventional method is based on the deep convolutional neural networks and became famous as the winner of the ImageNet Large Scale Visual Recognition Competition (ILSVRC) in 2012 [21].

Method #1 was used to classify the image data using the sharpening mask to emphasize the boundary of colors, while method #2 used the median filter to remove ambient noise pixels. The proposed algorithm involved the combined use of the sharpening mask and median filter for clear representation and removal of the noise pixels in the spectrogram.

Regarding the classification accuracy in the time division, Set A produced the highest percentage classification in comparison with other Sets. With increasing time length of the spectrogram, the percentage classification decreased when using the CNNs. This was because of the longer time spent in changing the noise environment, and the increased probability of error in the classification due to the reduced number of image data. The detailed confusion matrix of classification results is further analyzed in Section 5.2, below. Each number is the average of 10 classifications.

### 5.2. Data Analysis

Table 2 is a confusion matrix of classification results in the time length of Set A using the CNNs. The vertical noise numbers in the table represent the true class (Target Class), while the horizontal noise numbers represent the predicted class (Output Class). The numbers in the diagonal cells are the numbers of correct classifications, while those in the off-diagonal cells are the numbers of incorrect classifications. The percentage of correct classifications relative to the total number of observations are also shown for each noise number. The results reveal high classification accuracies irrespective of the use or type of filter. In addition, there are no significant differences between the spectrogram image classifications for the four different methods, because there was enough input data to classify, and the performance of the CNN was excellent. 

**Figure 4:**
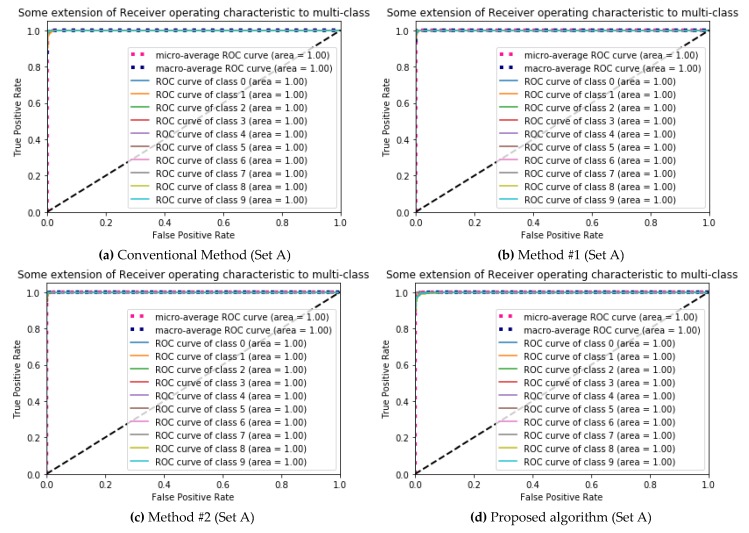
The Receiver Operating Characteristic (ROC) curve for Table 2: (**a**) Conventional Method; (**b**) Method #1, only the sharpening mask is applied; (**c**) Method #2, only the median filter is applied; (**d**) proposed algorithm, both the sharpening mask and the median filter are applied; the length of time of Set A is 1 s.

Figure 4 shows the Receiver Operating Characteristic (ROC) curve of multilabel classification for Table 2. The classification performance was confirmed through the ROC curves of all noises, which are close to the top and left-hand borders. All of the area under the ROC curve (AUC) were 1.0, meaning that the score describes the quality of the classification performance.

As can be seen from Table 3, when the environmental noises of the spectrogram image with a time length of Set B were classified using the four different methods, the classification accuracies were similar to those of Set A. In the cases of subway noise (N8) and traffic noise (N9), the classification rates using the sharpening mask and median filter were much better than when the filters were not used. 

Café noise (N1) was misclassified as subway noise (N8) and traffic noise (N9), respectively, because the irregular high-frequency noises in the café presented energy distributions similar to those of subway noise (N8) and traffic noise (N9). Subway noise (N8) was also misclassified as traffic noise (N9). Because the energy distributions of these two noises are similar, neither the sharpening mask (c) nor the median filter (d) produced significantly differing effects from them.

**Figure 5:**
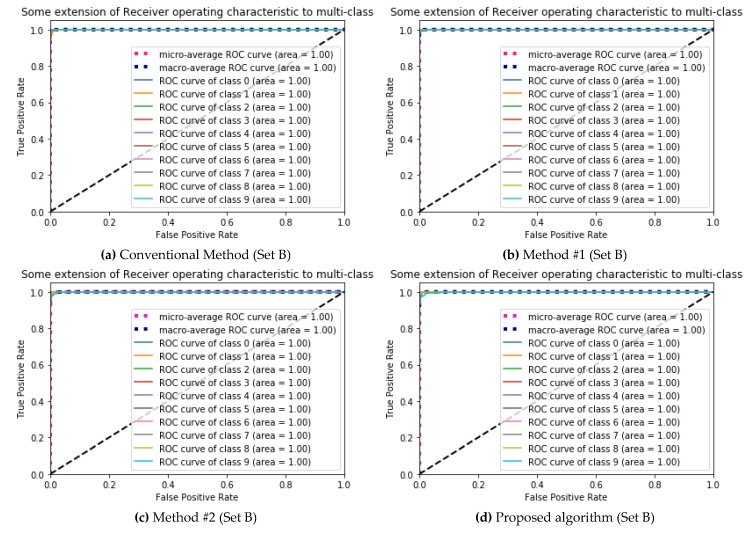
The Receiver Operating Characteristic (ROC) curve for Table 3: (**a**) Conventional Method; (**b**) Method #1, only the sharpening mask is applied; (**c**) Method #2, only the median filter is applied; (**d**) proposed algorithm, both the sharpening mask and the median filter are applied; the length of time of Set B is 2 s.

Figure 5 shows the Receiver Operating Characteristic (ROC) curve of multilabel classification for Table 3. The classification performance was confirmed through the ROC curves of all noises, which are close to the top and left-hand borders. All of the area under the ROC curve (AUC) were 1.0, meaning that the score describes the quality of the classification performance.

Table 4 presents the environmental noise classification results for a time length of Set C, which produced decreased image classification accuracies for some noise types compared with the classifications for Set A and B. Specifically, the classification accuracies when using the median filter (Method #2) were lower than the other results. This means that the characteristics and the distribution of noise could not be distinguished over longer time lengths because the median filter caused a smoothing effect. In Table 4c, café noise (N1) is incorrectly classified as subway noise (N8) and traffic noise (N9), and traffic noise (N9) s incorrectly classified as cafe noise (N1) and subway noise (N8), resulting in a reduced overall classification accuracy. Café noise (N1), traffic noise (N9) have similar energy distributions because they contain multiple voices in other complexed environments, with the sounds concentrated in the low-frequency range. In the case of café noise (N1), subway noise (N8) and traffic noise (N9), for which the conventional method produces relatively low classification accuracies, proposed algorithm affords significant improvements.

**Figure 6:**
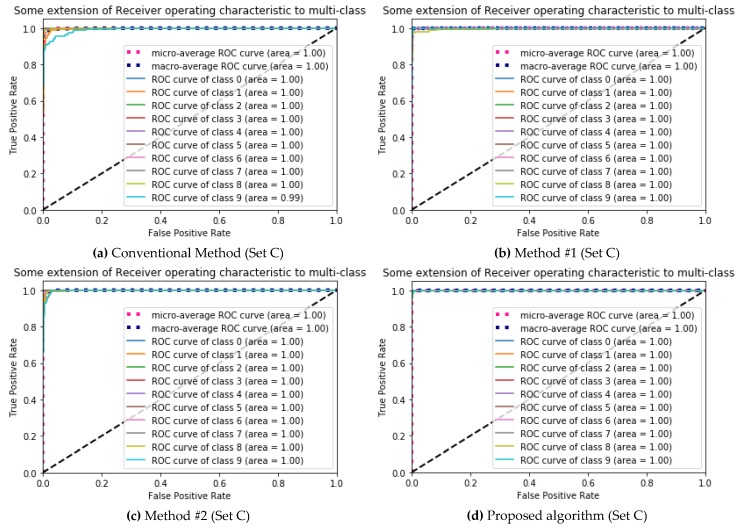
The Receiver Operating Characteristic (ROC) curve for Table 4: (**a**) Conventional Method; (**b**) Method #1, only the sharpening mask is applied; (**c**) Method #2, only the median filter is applied; (**d**) proposed algorithm, both the sharpening mask and the median filter are applied; the length of time of Set C is 4 s.

Figure 6 shows the Receiver Operating Characteristic (ROC) curve of multilabel classification for Table 4. The classification performance was confirmed through the ROC curves of all noises, which are close to the top and left-hand borders. All of the area under the ROC curve (AUC) were 0.99, meaning that the score describes the quality of the classification performance.

Table 5 presents the environmental noise classification results for a time length of Set D. Comparison of Table 5a,d shows that the proposed algorithm produces a 97.93% classification accuracy for cafe noise (N1), which is highly classified compared with the other methods. In addition, the classification accuracy of traffic noise (N9) with the proposed algorithm was also increased to 98.22%.

Overall, the proposed algorithm produces >96.4% classification accuracy for all environmental noises. That means the results show that the classification accuracy does not significantly decrease even for a time length of Set D when the two types of filters are applied to the input data of the CNNs. 

**Figure 7:**
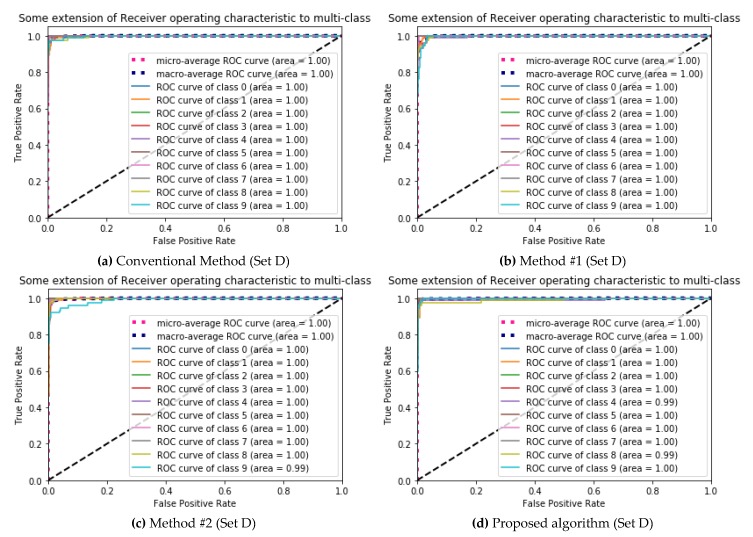
The Receiver Operating Characteristic (ROC) curve for Table 5: (**a**) Conventional Method; (**b**) Method #1, only the sharpening mask is applied; (**c**) Method #2, only the median filter is applied; (**d**) proposed algorithm, both the sharpening mask and the median filter are applied; the length of time of Set D is 8 s.

Figure 7 shows the Receiver Operating Characteristic (ROC) curve of multilabel classification for Table 5. The classification performance was confirmed through the ROC curves of all noises, which are close to the top and left-hand borders. All of the area under the ROC curve (AUC) were 1.0, meaning that the score describes the quality of the classification performance.

## 6. Conclusions

In this study, we proposed an algorithm for the classification of environmental noises in hearing aids and verified the performance. The proposed algorithm was to transform the sound data into image data for using as the input data of CNNs. The spectrogram images of the transformation were generated by dividing 10 environmental noises using four different time lengths, respectively, and the correct classification accuracies were compared for cases when a sharpening mask, median filter, and both were applied to the image data, respectively. We found that the correct noise classification accuracies for hearing aids using a CNNs gradually decreased with increasing time length of the spectrogram images due to the randomly changing noise characteristics. Regarding the type of filter used, the classification accuracy for the sharpening mask was higher than that for the median filter. In other words, it was more effective to sharpen the boundaries of the energy distribution in the spectrogram images than to remove the noise pixels from the images with obvious colors. Particularly, the combined use of the sharpening mask and median filter for a spectrogram time length of Set D increased the classification accuracy from 95.24% when no filter is used to 98.73%, which is comparable to the classification accuracy (98.79%) without a filter (conventional method) for a time length of Set A.

The proposed noise classification algorithm is thus effective for low computational complexity in long-term noise estimation and classification for hearing aids, as well as for environmental noise monitoring over a period of time, eliminating the need for real-time noise estimation. In addition, other types of filters that can clearly identify noise characteristics can be combined to further improve the use of CNNs for noise classification toward enhancing the performance of hearing aids.

## Figures and Tables

**Table 1:** Summary of the classification accuracy (%) applying different methods: (**a**) Conventional Method; (**b**) Method #1, only the sharpening mask is applied; (**c**) Method #2, only the median filter is applied; (**d**) proposed algorithm, both the sharpening mask the median filter are applied; the length of time of Set A is 1 s, Set B is 2 s, Set C is 4 s and Set D is 8 s.

**Test #**	**Set A**	**Set B**	**Set C**	**Set D**	**Test #**	**Set A**	**Set B**	**Set C**	**Set D**
	**(1 s)**	**(2 s)**	**(4 s)**	**(8 s)**		**(1 s)**	**(2 s)**	**(4 s)**	**(8 s)**
1	98.98	98.6	97.93	95.2	1	98.95	99.13	98.87	97.6
2	98.92	98.66	97.8	95.07	2	98.9	99.03	99	98.27
3	98.8	98.43	97.4	95.73	3	98.88	99	98.8	97.87
4	98.87	98.66	98.27	95.2	4	98.9	99.2	98.73	98
5	98.78	98.6	98.27	94	5	98.95	98.83	98.73	98.13
6	98.61	98.7	98.13	96.4	6	98.95	98.46	97.8	95.87
7	98.73	98.86	98.27	94.8	7	98.9	98.86	98.73	98.13
8	98.58	98.83	98.47	93.87	8	98.92	98.6	99.2	96.13
9	98.83	98.63	96.47	95.73	9	98.82	98.96	98.6	97.87
10	98.8	99.26	98.27	96.4	10	98.87	98.9	97.93	95.73
**AVG ^1^**	**98.79**	**98.72**	**97.93**	**95.24**	**AVG ^1^**	**98.9**	**98.9**	**98.64**	**97.36**
**SD ^2^**	**0.12**	**0.23**	**0.6**	**0.87**	**SD ^2^**	**0.04**	**0.23**	**0.44**	**1.02**
(**a**) Conventional Method(Spectrogram only)					(**b**) Method #1(Spectrogram + Sharpening Mask)				
**Test #**	**Set A**	**Set B**	**Set C**	**Set D**	**Test #**	**Set A**	**Set B**	**Set C**	**Set D**
	**(1 s)**	**(2 s)**	**(4 s)**	**(8 s)**		**(1 s)**	**(2 s)**	**(4 s)**	**(8 s)**
1	99.12	99.06	96.2	95.47	1	99.37	99.13	99.13	99.07
2	99.12	98.83	96	97.47	2	99.23	99.16	99.33	98.93
3	99.07	99	96.07	96.13	3	99.25	99.2	99.33	98.67
4	99.15	98.83	96.53	96.27	4	99.35	99.23	99.27	98.67
5	98.95	98.53	96.13	95.33	5	99.42	99.26	99.13	99.2
6	99.05	97.99	95.87	94.27	6	99.2	99.26	98.93	97.73
7	98.95	99.03	95.53	97.6	7	98.97	98.5	98.67	98.8
8	99.07	98.63	97.53	97.07	8	99.38	98.83	99.47	98.53
9	98.93	99.16	98	95.33	9	99.07	99.33	98.8	99.2
10	98.97	99	97.27	95.73	10	99.28	99.43	98.87	98.53
**AVG ^1^**	**99.04**	**98.81**	**96.51**	**96.07**	**AVG ^1^**	**99.25**	**99.13**	**99.09**	**98.73**
**SD ^2^**	**0.08**	**0.35**	**0.81**	**1.06**	**SD ^2^**	**0.14**	**0.27**	**0.27**	**0.43**
(**c**) Method #2(Spectrogram + Median Filter)					(**d**) Proposed algorithm(Spectrogram + Sharpening Mask + Median Filter)				

^1^ AVG: Average of classification accuracies. ^2^ SD: Standard Deviation of classification accuracies.

**Table 2:** Summary of the classification accuracy (%) applying different methods in Set A: (**a**) Conventional Method; (**b**) Method #1, only the sharpening mask is applied; (**c**) Method #2, only the median filter is applied; (**d**) proposed algorithm, both the sharpening mask and the median filter are applied; the length of time of Set A is 1 s.

	**N0**	**N1**	**N2**	**N3**	**N4**	**N5**	**N6**	**N7**	**N8**	**N9**	**ACC**		**N0**	**N1**	**N2**	**N3**	**N4**	**N5**	**N6**	**N7**	**N8**	**N9**	**ACC**
											**(%)**												**(%)**
N0	599	0	0	0	0	0	0	0	0	0	**100**	N0	599	0	0	0	0	0	0	0	0	0	**100**
N1	0	582.8	0	0.2	0.8	0.4	4	0.9	4.3	5.6	**97.29**	N1	0	584.1	0	0.2	1	1.1	3.9	1.2	2.8	4.7	**97.51**
N2	0	0	597.7	0	0	0	0	0	0.4	0.9	**99.78**	N2	0	0	597.6	0	0	0	0	0	1.1	0.3	**99.76**
N3	0	0	0	599	0	0	0	0	0	0	**100**	N3	0	0	0	598.8	0	0	0	0	0	0.2	**99.96**
N4	0	0.3	0.1	0.2	594.1	0.4	1.1	0.1	0.3	2.2	**99.18**	N4	0	0.9	0	0	593.6	0.4	1.1	0.8	1.1	1.1	**99.09**
N5	0.1	0.3	0	0	0.2	593.1	4	0.3	0	0.9	**99.02**	N5	0.1	0.3	0	0	0	593.4	4.1	0.7	0	0.3	**99.07**
N6	0	3.4	0	0.3	0	4.3	587.3	0.8	0	2.8	**98.05**	N6	0	2.3	0	0	0	3.4	591.1	0.9	0	1.2	**98.68**
N7	0	0.8	0	0	0	0	0.1	596.8	0.3	1	**99.63**	N7	0	0.8	0	0	0	0.2	0.2	595.7	0	2	**99.46**
N8	0	9.7	0.1	0	0.1	0.7	0	0	585.9	2.6	**97.81**	N8	0	8.3	0	0	0	1.1	0	0	585.8	3.9	**97.77**
N9	0.2	8	0.1	0.1	0.4	3.1	1.3	0.4	3.3	581.9	**97.14**	N9	0.2	5.9	0.7	0	0.3	2.8	1.2	0.4	2.6	584.9	**97.64**
	**Average Classification Accuracy**										**98.79**		**Average Classification Accuracy**										**98.9**
(**a**) Conventional Method (Set A)												(**b**) Method #1 (Set A)											
	**N0**	**N1**	**N2**	**N3**	**N4**	**N5**	**N6**	**N7**	**N8**	**N9**	**ACC**		**N0**	**N1**	**N2**	**N3**	**N4**	**N5**	**N6**	**N7**	**N8**	**N9**	**ACC**
											**(%)**												**(%)**
N0	599	0	0	0	0	0	0	0	0	0	**100**	N0	599	0	0	0	0	0	0	0	0	0	**100**
N1	0	581.9	0	0.3	0.4	0.9	5.9	1.1	3.4	5	**97.14**	N1	0	586.1	0	0.2	0.6	0.6	3.4	0.6	3.9	3.7	**97.85**
N2	0	0	598.6	0	0	0	0	0	0.1	0.3	**99.93**	N2	0	0	598.1	0	0	0	0	0	0.6	0.3	**99.85**
N3	0	0	0	598.9	0	0	0	0	0	0.1	**99.98**	N3	0	0.1	0	598.9	0	0	0	0	0	0	**99.98**
N4	0	0.9	0	0	595.3	0.8	0.8	0.2	0.8	0.2	**99.39**	N4	0	0.6	0	0	597	0	0	0.2	0.4	0.2	**99.67**
N5	0.1	0.4	0	0	0.1	594.6	3.2	0.4	0	0.1	**99.26**	N5	0	0.1	0	0	0	595.4	2.8	0	0	0.7	**99.41**
N6	0	1.4	0	0.3	0	2.7	592.8	1	0	0.8	**98.96**	N6	0	1.3	0	0.2	0	2.8	593	1.4	0	0.2	**99**
N7	0	0.9	0	0	0.1	0	0.1	597.2	0	0.7	**99.7**	N7	0	0.4	0	0	0	0	0.1	597.8	0	0.7	**99.8**
N8	0	6.4	0.1	0	0.1	0.4	0	0	589	2.9	**98.33**	N8	0	5.9	0	0	0.2	0.4	0	0	590.7	1.8	**98.61**
N9	0	6.2	0.3	0	1.3	2	1.2	0.1	2.2	585.6	**97.76**	N9	0	4.9	0.3	0	0.6	1.3	1.1	0.1	1.8	588.9	**98.31**
	**Average Classification Accuracy**										**99.04**		**Average Classification Accuracy**										**99.25**
(**c**) Method #2 (Set A)												(**d**) Proposed algorithm (Set A)											

Note: the correct classifications in the diagonal cells and especially the incorrect classifications in the rest to be express in different colors.

**Table 3:** Summary of the classification accuracy (%) applying different methods in Set B: (**a**) Conventional Method; (**b**) Method #1, only the sharpening mask is applied; (**c**) Method #2, only the median filter is applied; (**d**) proposed algorithm, both the sharpening mask and the median filter are applied; the length of time of Set B is 2 s.

	**N0**	**N1**	**N2**	**N3**	**N4**	**N5**	**N6**	**N7**	**N8**	**N9**	**ACC**		**N0**	**N1**	**N2**	**N3**	**N4**	**N5**	**N6**	**N7**	**N8**	**N9**	**ACC**
											**(%)**												**(%)**
N0	299	0	0	0	0	0	0	0	0	0	**100**	N0	299	0	0	0	0	0	0	0	0	0	**100**
N1	0	289	0	0.2	0.9	0.6	1.3	1	2.8	3.2	**96.66**	N1	0	293.1	0	0	0.2	0.9	0.9	0.9	0.9	2.1	**98.03**
N2	0	0	298.2	0	0	0	0	0	0	0.8	**99.74**	N2	0	0	298.4	0	0	0	0	0	0.1	0.4	**99.81**
N3	0	0.1	0	298.9	0	0	0	0	0	0	**99.96**	N3	0	0.2	0	298.4	0	0	0	0	0	0.3	**99.81**
N4	0	0.2	0.1	0	294.3	0.2	0.4	0.9	1.3	1.4	**98.44**	N4	0	0	0.2	0	295	0	0.8	0	0.3	2.7	**98.66**
N5	0	0	0	0	0	297.2	1.6	0	0	0.2	**99.41**	N5	0.2	0.7	0	0	0	296.3	1.3	0	0	0.4	**99.11**
N6	0	0.2	0	0	0	1.8	297	0	0	0	**99.33**	N6	0	0.3	0	0	0	1	296.2	0.3	0	1.1	**99.07**
N7	0	0.4	0	0	0	0	0.1	298.4	0	0	**99.81**	N7	0	0	0	0	0	0	0.2	298.7	0	0.1	**99.89**
N8	0	4.9	0	0	0.4	0.4	0	0	291.6	1.7	**97.51**	N8	0	3.3	0	0	0.8	1.1	0	0	290	3.8	**96.99**
N9	0	4.1	0.8	0.1	0.6	0.7	2	0.6	2.2	288	**96.32**	N9	0	1.7	0	0	0.7	1.7	1.1	0.8	1.1	292	**97.66**
	**Average Classification Accuracy**										**98.72**		**Average Classification Accuracy**										**98.9**
(**a**) Conventional Method (Set B)												(**b**) Method #1 (Set B)											
	**N0**	**N1**	**N2**	**N3**	**N4**	**N5**	**N6**	**N7**	**N8**	**N9**	**ACC**		**N0**	**N1**	**N2**	**N3**	**N4**	**N5**	**N6**	**N7**	**N8**	**N9**	**ACC**
											**(%)**												**(%)**
N0	299	0	0	0	0	0	0	0	0	0	**100**	N0	299	0	0	0	0	0	0	0	0	0	**100**
N1	0	292.3	0	0	0.2	0.8	1.2	0.6	2.7	2.1	**97.77**	N1	0	291	0	0.2	0.3	0.4	1.2	0	2.6	3.2	**97.32**
N2	0	0	298.9	0	0	0	0	0	0	0.1	**99.96**	N2	0	0	298.8	0	0	0	0	0	0	0.2	**99.93**
N3	0	0	0	298.8	0.2	0	0	0	0	0	**99.93**	N3	0	0.1	0	298.7	0.1	0	0	0	0	0.1	**99.89**
N4	0	0.6	0.2	0	295.9	0	0.2	0.2	0.4	1.4	**98.96**	N4	0	0.1	0.1	0	296.4	0.1	0.8	0	0.3	1.1	**99.15**
N5	0	0.4	0	0	0	296.2	2.1	0	0.1	0.1	**99.07**	N5	0	0.4	0	0	0	297.4	1.1	0	0	0	**99.48**
N6	0	0.2	0.1	0.1	0.1	2.7	295.3	0.2	0	0.2	**98.77**	N6	0	0.3	0	0	0	1.8	296.3	0.2	0	0.3	**99.11**
N7	0	0.2	0	0	0	0	0	298.6	0.1	0.1	**99.85**	N7	0	0.1	0	0	0	0	0	298.3	0.1	0.4	**99.78**
N8	0	6.4	0	0	0.4	0.1	0	0	289.7	2.3	**96.88**	N8	0	2	0	0	0.2	0.3	0	0	294.2	2.2	**98.4**
N9	0	5.4	0.3	0	0.4	0.7	0.6	0	1.8	289.8	**96.92**	N9	0.1	2.2	0.1	0	0.4	0.6	0.3	0.2	1.3	293.7	**98.22**
	**Average Classification Accuracy**										**98.81**		**Average Classification Accuracy**										**99.13**
(**c**) Method #2 (Set B)												(**d**) Proposed algorithm (Set B)											

Note: the correct classifications in the diagonal cells and especially the incorrect classifications in the rest to be express in different colors.

**Table 4:** Summary of the classification accuracy (%) applying different methods in Set C: (**a**) Conventional Method; (**b**) Method #1, only the sharpening mask is applied; (**c**) Method #2, only the median filter is applied; (**d**) proposed algorithm, both the sharpening mask and the median filter are applied; the length of time of Set C is 4 s.

	**N0**	**N1**	**N2**	**N3**	**N4**	**N5**	**N6**	**N7**	**N8**	**N9**	**ACC**		**N0**	**N1**	**N2**	**N3**	**N4**	**N5**	**N6**	**N7**	**N8**	**N9**	**ACC**
											**(%)**												**(%)**
N0	150	0	0	0	0	0	0	0	0	0	**100**	N0	150	0	0	0	0	0	0	0	0	0	**100**
N1	0	144.7	0	0	0	0.4	0.8	0.4	2	1.7	**96.44**	N1	0	146.4	0	0	0.1	0.3	0.8	0.1	0.3	1.9	**97.63**
N2	0	0	149.1	0	0	0	0	0	0.3	0.6	**99.41**	N2	0	0	149.6	0	0	0	0	0	0.2	0.2	**99.7**
N3	0	0	0	149.7	0.3	0	0	0	0	0	**99.78**	N3	0	0	0	150	0	0	0	0	0	0	**100**
N4	0	0.6	0.3	0.1	145.7	0	0.2	0.9	0.7	1.6	**97.11**	N4	0	0.6	0	0.1	146.3	0.2	0.7	0.2	0.4	1.4	**97.56**
N5	0	0	0	0	0.1	149.7	0.1	0	0	0.1	**99.78**	N5	0	0	0	0	0	149.4	0.6	0	0	0	**99.63**
N6	0	0.6	0	0	0.1	2.8	146.2	0.3	0	0	**97.48**	N6	0	0	0	0	0	1	148.9	0	0	0.1	**99.26**
N7	0	0	0	0	0	0	0.1	149.8	0	0.1	**99.85**	N7	0	0	0	0	0	0	0	150	0	0	**100**
N8	0	2.4	0.6	0	0	0	0	0.2	145.2	1.6	**96.81**	N8	0	4.3	0	0	0.1	0	0	0	144.1	1.4	**96.07**
N9	0.2	7.4	0	0.1	0.2	0.6	0	0.6	2	138.9	**92.59**	N9	0	2.6	0	0.1	0.3	0.8	0.8	0.1	0.4	144.9	**96.59**
	**Average Classification Accuracy**										**97.93**		**Average Classification Accuracy**										**98.64**
(**a**) Conventional Method (Set C)												(**b**) Method #1 (Set C)											
	**N0**	**N1**	**N2**	**N3**	**N4**	**N5**	**N6**	**N7**	**N8**	**N9**	**ACC**		**N0**	**N1**	**N2**	**N3**	**N4**	**N5**	**N6**	**N7**	**N8**	**N9**	**ACC**
											**(%)**												**(%)**
N0	150	0	0	0	0	0	0	0	0	0	**100**	N0	150	0	0	0	0	0	0	0	0	0	**100**
N1	0	136.2	0	0	0.7	0.2	2.6	0.7	3.6	6.1	**90.81**	N1	0	147.4	0	0	0.2	0	0.6	0.1	0.7	0.9	**98.37**
N2	0	0	148.8	0	0.1	0	0	0	0.6	0.6	**99.19**	N2	0	0	149.6	0	0	0	0	0	0.2	0.2	**99.7**
N3	0	0	0	149.8	0.2	0	0	0	0	0	**99.85**	N3	0	0	0	149.9	0.1	0	0	0	0	0	**99.93**
N4	0	0.3	0.3	1.8	142.7	0	0.6	0.3	2.2	1.8	**95.11**	N4	0	0.3	0.1	0.2	147.1	0	0.2	0.3	0.1	1.1	**98.37**
N5	0	0.4	0	0	0	148.6	0.8	0	0.2	0	**99.04**	N5	0	0	0	0	0	149.4	0.6	0	0	0	**99.63**
N6	0	0.6	0	0.2	0	3.4	145.1	0.3	0	0.3	**96.74**	N6	0.1	0	0	0.1	0	1.4	147.9	0.1	0	0.1	**98.74**
N7	0	0.1	0	0	0	0	0.1	148.8	0.1	0.9	**99.19**	N7	0	0.1	0	0	0	0	0	149.7	0	0.2	**99.78**
N8	0	4.8	0	0	0.4	0.2	0	0.2	141.8	2.6	**94.52**	N8	0	1.7	0	0	0.1	0.1	0	0	147.1	0.7	**98.3**
N9	0	5.4	1.2	0.6	0.2	0.2	1.8	0.8	3.8	136	**90.67**	N9	0	1.4	0	0.1	0.3	0.1	0.3	0.3	0.2	146.9	**98.07**
	**Average Classification Accuracy**										**96.51**		**Average Classification Accuracy**										**99.09**
(**c**) Method #2 (Set C)												(**d**) Proposed algorithm (Set C)											

Note: the correct classifications in the diagonal cells and especially the incorrect classifications in the rest to be express in different colors.

**Table 5:** Summary of the classification accuracy (%) applying different methods in Set D: (**a**) Conventional Method; (**b**) Method #1, only the sharpening mask is applied; (**c**) Method #2, onyl the median filter is applied; (**d**) proposed algorithm, both the sharpening mask and the median filter are applied; the length of time of Set D is 8 s.

	**N0**	**N1**	**N2**	**N3**	**N4**	**N5**	**N6**	**N7**	**N8**	**N9**	**ACC**		**N0**	**N1**	**N2**	**N3**	**N4**	**N5**	**N6**	**N7**	**N8**	**N9**	**ACC**
											**(%)**												**(%)**
N0	75	0	0	0	0	0	0	0	0	0	**100**	N0	75	0	0	0	0	0	0	0	0	0	**100**
N1	0	64.6	0	0	0.3	0.4	1.4	0.1	3.2	4.9	**86.07**	N1	0	69.3	0	0	0	0	2.3	0	1.9	1.4	**92.44**
N2	0	0	74.2	0	0	0	0	0	0.1	0.7	**98.96**	N2	0	0	74.9	0	0	0	0	0	0	0.1	**99.85**
N3	0	0	0	74.6	0.4	0	0	0	0	0	**99.41**	N3	0	0	0	74.9	0.1	0	0	0	0	0	**99.85**
N4	0	0.3	0.1	0	71.6	0.2	0	0.6	0.4	1.8	**95.41**	N4	0	0	0.4	0.2	70.8	0.2	0.6	0.4	0.3	2	**94.37**
N5	0	0.1	0	0	0	74.3	0.3	0	0.1	0.1	**99.11**	N5	0	0	0	0	0	74.9	0.1	0	0	0	**99.85**
N6	0	0	0	0	0	4.2	70	0.1	0	0.7	**93.33**	N6	0	0	0	0	0	0.7	74.3	0	0	0	**99.11**
N7	0	0	0	0	0	0.1	0	74.3	0	0.6	**99.11**	N7	0	0	0	0	0	0	0.1	74.9	0	0	**99.85**
N8	0	2.1	0	0	2	0.2	0	0	69.2	1.4	**92.3**	N8	0	2.1	0	0	0.3	0.1	0.1	0	70.6	1.8	**94.07**
N9	0	3.7	0.1	0.1	0.1	0.6	0	0.6	3.3	66.6	**88.74**	N9	0	2	0	0	0.7	0.6	0.1	0.3	0.7	70.7	**94.22**
	**Average Classification Accuracy**										**95.24**		**Average Classification Accuracy**										**97.36**
(**a**) Conventional Method (Set D)												(**b**) Method #1 (Set D)											
	**N0**	**N1**	**N2**	**N3**	**N4**	**N5**	**N6**	**N7**	**N8**	**N9**	**ACC**		**N0**	**N1**	**N2**	**N3**	**N4**	**N5**	**N6**	**N7**	**N8**	**N9**	**ACC**
											**(%)**												**(%)**
N0	75	0	0	0	0	0	0	0	0	0	**100**	N0	75	0	0	0	0	0	0	0	0	0	**100**
N1	0	66.4	0	0	1	0.1	1.8	0	1.8	3.9	**88.59**	N1	0	73.3	0	0	0.1	0.1	0.4	0	0.2	0.8	**97.78**
N2	0	0	74.1	0	0	0	0	0	0	0.9	**98.81**	N2	0	0	74.7	0	0	0	0	0	0.1	0.2	**99.56**
N3	0	0	0	74.8	0.2	0	0	0	0	0	**99.7**	N3	0	0	0	74.8	0.2	0	0	0	0	0	**99.7**
N4	0	0.2	0.3	1.1	70.7	0.2	0.3	0	0.7	1.4	**94.22**	N4	0	0.4	0.3	0	72.9	0.1	0	0	0.7	0.6	**97.19**
N5	0	0	0	0	0	74.6	0.4	0	0	0	**99.41**	N5	0	0	0	0	0	74.9	0.1	0	0	0	**99.85**
N6	0	0	0	0	0	2.4	72.4	0	0	0.1	**96.59**	N6	0	0	0	0	0	0.8	74.2	0	0	0	**98.96**
N7	0	0	0	0	0	0	0.4	74.2	0	0.3	**98.96**	N7	0	0	0	0	0	0	0.1	74.9	0	0	**99.85**
N8	0	1.3	0.1	0	0.4	0.1	0	0	71	2	**94.67**	N8	0	1.1	0	0	0.2	0.3	0	0	72.6	0.8	**96.74**
N9	0	1.8	1.7	0.3	1	0.4	1.6	0.4	0.4	67.3	**89.78**	N9	0	0.8	0	0	0.1	0.4	0.3	0.1	0	73.2	**97.63**
	**Average Classification Accuracy**										**96.07**		**Average Classification Accuracy**										**98.73**
(**c**) Method #2 (Set D)												(**d**) Proposed algorithm (Set D)											

Note: the correct classifications in the diagonal cells and especially the incorrect classifications in the rest to be express in different colors.
